# Building Diversity, Equity, Inclusion, and Accessibility Capacity: Resources to Promote Best Practices Among Professionals in Scholarly Publishing

**DOI:** 10.5888/pcd20.230332

**Published:** 2023-11-22

**Authors:** Leonard Jack, Peter J. Olson, Patricia K. Baskin, Otito F. Iwuchukwu

**Affiliations:** 1 Preventing Chronic Disease, Office of Medicine and Science, National Center for Chronic Disease Prevention and Health Promotion, Centers for Disease Control and Prevention, Atlanta, Georgia; 2 JAMA Network, Chicago, Illinois; 3 American Academy of Neurology, Minneapolis, Minnesota; 4 Fairleigh Dickinson University School of Pharmacy and Health Sciences, Teaneck, New Jersey

This article is part of a joint publication initiative between *Science Editor* and *Preventing Chronic Disease.* *Science Editor *is the primary publisher, while *Preventing Chronic Disease *is the secondary publisher.

The Council of Science Editors (CSE) is an international organization of more than 500 editorial professionals in the scientific, scientific publishing, and information science communities. The organization’s goal is to serve as an authoritative resource on current and emerging issues in the communication of scientific information (1). Similar to other scholarly publishing organizations, CSE continues to facilitate important conversations and training regarding why, how, and where principles of diversity, equity, inclusion, and accessibility (DEIA) should be integrated into scholarly publishing. With guidance from CSE members with expertise in DEIA in scholarly publishing, and the approval of CSE’s Board of Directors, the organization established the DEI Committee in 2021 (which was expanded to the DEIA Committee in 2023). The purpose of the DEIA Committee is “to support the organization in building capacity among its leadership, members, and the profession at large to deliver programmatic activities and training that integrate [DEIA] best practices in science editing, publication management, scholarly publishing and communication, member recruitment, participation, and engagement” (2).

Since the committee’s inception, CSE has implemented and/or participated in 8 broad-ranging DEIA-related activities: 1) adding new content to CSE’s *Recommendations for Promoting Integrity in Scientific Journal Publications* (3) related to DEIA best practices in scholarly publishing; 2) completing a DEIA sensitivity review of *Scientific Style and Format* (4), the CSE style manual, for its upcoming 9th edition, scheduled for publication in 2024; 3) a DEIA-related symposium to update members on CSE’s progress in achieving DEIA-related objectives and activities identified in CSE’s Strategic Plan (2); 4) establishing a DEIA column in *Science Editor* (5), CSE’s quarterly magazine; 5) implementing an inaugural 1-day DEIA short course to a range of professionals in scholarly publishing; 6) implementing its Ethics Clinic on Diversity, Equity, and Inclusion (6); 7) actively serving as a member organization for the Coalition for Diversity & Inclusion in Scholarly Communications (C4DISC) (3); and 8) establishing CSE’s DEIA Scholarly Resources web page (7).

Collectively, these activities help publishers, editorial leadership of journals, journal editors, editorial teams, peer reviewers, and authors gain access to numerous resources, tools, educational materials, and training opportunities. This access allows scholarly publishing professionals to develop the knowledge, skills, and abilities to increase equitable participation and decision-making among diverse groups of individuals, increase trust among stakeholders, encourage civility, and support fairness throughout all aspects of the industry. The road to fully realizing equity, diversity, inclusion, and accessibility requires persistent attention, collaboration, and introspection from professionals and organizations in both the early stages (eg, deciding who to invite to the table) and more advanced stages (eg, deciding to use data collected to revise policies and procedures) of that realization (8).

Fortunately, there are several published and publicly available DEIA-related resources to assist professionals working in the field of scholarly publishing. The formation of CSE’s DEIA Scholarly Resources web page (7) has yielded a one-stop repository that provides access to resources in 7 categories: 1) DEIA Committees of Trade and Professional Organizations in Scholarly Publishing; 2) DEIA and Peer Review; 3) DEIA Statements and Policies from Journals, Trade and Professional Associations, and Publishers; 4) Bias, Discrimination, and Racism; 5) Data Collection on Diversity, Equity, Inclusion, and Accessibility; 6) Reporting Sex, Gender, and Race in Publications; and 7) Inclusive Language Communication (Figure).

**Figure:**
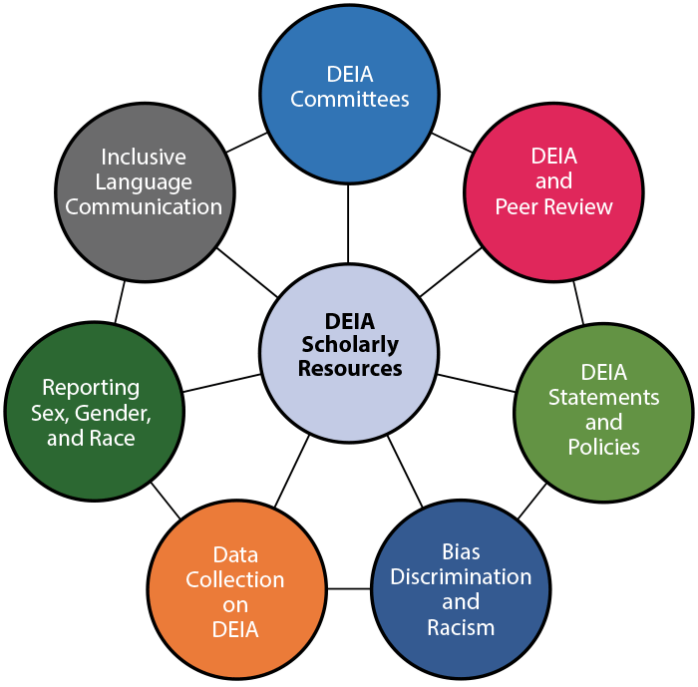
Council of Science Editors’ 7 categories of diversity, equity, inclusion, and accessibility scholarly resources. Abbreviation: DEIA diversity, equity, inclusion, and accessibility.

The resources in these 7 categories are briefly described below and were identified through various approaches, including literature searches, resources identified by CSE DEIA members, and resources submitted via the web page from CSE members. Resources identified for inclusion ranged from peer-reviewed papers, position statements, guidance documents, online training webinars, and more. The content provided within each of the 7 categories is by no means comprehensive, and the inclusion of a particular resource does not necessarily represent endorsement by CSE or the Centers for Disease Control and Prevention. As stated on CSE’s website: “The categories are broad, and information may overlap more than one category” (7).

## 1. DEIA Committees of Trade and Professional Organizations in Scholarly Publishing

Various trade and professional associations within the scholarly publishing arena are committed to addressing issues of diversity, equity, inclusion, and accessibility in their communities (9). They have formed coalitions and committees to provide resources to individuals and organizations that provide information about DEIA-related activities such as training, networking, changing organizational culture, and use of best practices in language usage, editing, and organization of editorial boards and teams.

Resources in this category describe the formation of DEIA-related committees of trade and professional organizations in scholarly publishing as well as their purposes, composition, and activities undertaken by their membership. Activities undertaken by DEIA committees include establishing toolkits for allies and organizations, establishing guidelines for inclusive language and images, implementing a series of discussion sessions to share knowledge solutions for accessibility, disseminating strategies to diversify editorial boards, and ways to improve the peer review process and the content of published articles. These resources also describe ways in which DEIA committees collaborate with their organizations’ leadership and other organizational committees to ensure that DEIA best practices are used in creating program and conference planning, identifying speakers, recruiting members, and promoting unbiased science editing among the member journals. In addition, outside of their own organizations, DEIA committees describe ways in which they have worked collectively to assist in planning seminars, educational opportunities, and other events for the field of scholarly publishing in general.

## 2. DEIA and Peer Review

Journals depend on subject area experts to help assess the strengths and weaknesses of content submitted for consideration (10). Journals are expected to continuously review guidance provided to peer reviewers to help minimize biases that may occur during the review process. This guidance is necessary to help scholarly journals screen and provide authors with feedback that positions those journals to disseminate evidence-based, culturally appropriate, trustworthy content. The resources found in this category provide information about implementing strategies to overcome inequities that may be unintentionally built into the peer review process. If not addressed, these inequities could result in publishing content that lacks rigor and credibility.

The content in this category offers examples from journals and publishers that present ways to diversify volunteer groups that comprise editorial boards, associate editors, and peer reviewers. Several resources provide insight into how journals can establish commitments through policies and procedures that encourage best practices in developing diverse, equitable, and inclusive peer review teams. Resources are also available that provide examples on how to collect demographic data to track diversity, how to use such data to monitor progress, and, if necessary, how to identify when it is necessary to implement a shift in approaches to address areas requiring improvement.

## 3. DEIA Statements and Policies From Journals, Trade and Professional Associations, and Publishers

It’s all well and good to say that you believe in the principles of DEIA; it’s another thing to commit to them. In recent years, many journals, trade organizations, professional associations, and publishers have not only acknowledged the need to advance DEIA practices within their institutions but also have published position statements or publicly declared their intent to do so (11). This section of the DEIA Scholarly Resources page provides access to published statements on developing DEIA frameworks; guiding principles for addressing bias based on race and ethnicity, gender, religion, disability, and other aspects of self-identification; DEIA-based editorial policies; and strategies to cultivate organizational practices that promote inclusion and diversity.

These statements and declarations go beyond abstract advocacy of DEIA principles. They are prudently crafted, comprehensive statements or declarations that describe demonstrable initiatives, communicate immediate policy updates, and divulge detailed progress reports. Many of these resources cite supportive data to substantiate the need for — and the potential impact of — actions designed to dismantle systemic bias, discrimination, and racism within an organization’s infrastructure. Many take the additional step of including links to ancillary resources that provide further guidance for implementing DEIA-related practices that can inspire institutional change.

## 4. Bias, Discrimination, and Racism

Systemic inequity is a deep-seated, pervasive concept that has been responsible for immeasurable societal historical challenges that persist to this day (12). These challenges are no less prevalent or problematic in scholarly publishing; fortunately, many scholarly publishing entities are taking bold steps forward to address the systemic frameworks of bias, discrimination, and racism that exist in every corner of the industry (13). This collection of resources includes large publishers that have codified their commitment to inclusion and equity and the elimination of systemic racism in scientific research and health affairs.

The resources in this category provide important information and perspectives on how bias, discrimination, and racism can permeate an institution’s infrastructure, its editorial processes, and any content it may produce and/or publish. Additionally, they reflect the difficulty of having discussions and asking the questions that will actionably address the existence of bias, racism, and discrimination within the scholarly publishing industry, including: How do bias, racism, and discrimination negatively impact a journal’s and/or a publisher’s ability to avoid publishing content that perpetuates historical harm? What opportunities exist for diverse participation on volunteer boards? What are the persistent representational issues in scholarly publishing?

Many of these resources offer pragmatic examples of how an institution can address and avoid systemically hierarchical, discriminatory, and biased frameworks within its operations and redefine its best practices to ensure both equity and integrity in its mission to publish scientific content. In addition, they offer examples of what journals in medicine, health care, pediatrics, and genomics are doing to establish an antiracist posture in scientific publication. Many of these statements offered by journals are geared toward establishing commitments to taking proactive steps to establish an antiracist future in scholarly publishing.

## 5. Data Collection on Diversity, Equity, Inclusion, and Accessibility

Documented lack of diverse representation among editorial leadership, editorial boards, peer reviewers, and authors remains a major barrier to achieving the aims of advancing DEIA principles in scholarly publishing (14). There are increasing expectations for publishers and journals to not only identify, implement, and publish plans to advance DEIA-related activities but also to increase accountability by collecting, using, and publishing demographic data of its editorial boards, peer reviewers, and authors (15). Collecting demographic data can provide key metrics useful in understanding who is deciding what is published (16). There may also be instances when it is deemed helpful to collect demographic data regarding organizational leadership and staff composition (16). This category provides information and resources on the importance of collecting demographic data, tools for collecting those data, and ways to use it to guide strategic planning and day-to-day decision-making. The resources in this category provide examples of ways journals can collect demographic questions during manuscript submission; in addition, they discuss barriers to the collection of demographic data and strategies to overcome those barriers.

## 6. Reporting Sex, Gender, and Race in Publications

Historical harm from faulty studies can be caused by many factors, including limited and unchallenged research questions, flawed methodological approaches, misaligned statistical testing and reporting, and unaddressed/uncorrected publication bias (17). As a result, journals have moved to publishing author guidance requiring greater reporting details on sex, gender, race, and ethnicity in published papers. This category provides examples of such journal guidance to authors to improve research integrity and transparency. Several resources are available, ranging from a checklist for reporting race and ethnicity in medical and science journals to reporting classification variables for individual characteristics (eg, race, indigeneity, national origin, gender, sexual orientation, and socioeconomic status). These resources discuss the importance of including an explanation in published articles of who identified participants’ classification, stating the sources of the classifications (eg, self-report, investigator observation, database, electronic health record, survey instrument), and the reasoning behind using race and ethnicity categories. The resources in this category continue to emphasize that journals must remain vigilant in updating guidance to authors to prevent methods and statistical analyses from being poorly described, making it difficult to replicate research and prove or disprove hypotheses or findings.

## 7. Inclusive Language Communication

Inclusive language is evolving at an unprecedented rate (18). Acceptable usage of certain terms, phrases, or concepts are constantly being reassessed, and many publishers and journals are diligently — and frequently — incorporating requisite updates regarding inclusive and nonbinary language into their style manuals and author guidelines (19). The resources in this category provide invaluable information and guidance from several prominent and well-respected institutions within the scholarly publishing industry, all of which are at the forefront of the effort to promote the use of language that is respectful toward authors, study participants, and readers alike.

An important ripple effect of this effort is that authors are more likely to feel included, invited, and motivated to submit their work to publications that have clearly demonstrated a commitment to inclusivity and sensitivity in scientific reporting. From policy toolkits to editorial style recommendations to study methodology guidelines, these resources stand at the cutting edge of inclusive language conventions and best practices, and the institutions that developed them continue to play a critical role in advancing and keeping pace with this ever-evolving aspect of the scientific enterprise.

## Conclusion

As with any field of interest, the number of DEIA-related resources continues to increase as publishers and journals learn from and build upon previously released guidance. As such, resources on CSE’s DEIA Scholarly Resources web page will continue to increase. These resources can assist journals at various stages of implementation of DEIA-centered activities. Journals, editorial offices, and publishers are in the best position to decide which resources are of best use and why. Determining where to start requires asking important and often difficult questions, including: Is there an understanding of why DEIA in scholarly publishing is important? Are there groups, networks, organizations, experts, or other stakeholders who are willing and available to engage and ask questions about where best to get started? Are there training opportunities available that can help increase knowledge, skills, and abilities among leadership, decision-makers, staff, and volunteers? The key is to identify a reasonable plan of action, encourage participation from a wide range of stakeholders, and adopt patience to execute the plan over time.

The CSE DEIA Committee has envisioned a process of curating and maintaining the Scholarly Resources web page over time to provide access to current content housed in one location. The committee encourages visitors not only to explore the contents and their evolution, but also to consider submitting resources for inclusion as they become available. Authors, journals, creators of training opportunities, and other scholarly publishing professionals are encouraged to submit requests (20) to include resources for consideration in any of the 7 categories.
